# Thirty-Day Outcomes in Ultrasound Versus CT-Only Imaging in Obese Adults With Acute Appendicitis

**DOI:** 10.7759/cureus.116517

**Published:** 2026-09-18

**Authors:** Joseph McKinnerney, Jason Yan, Harpreet Grewal, Vivek Joshi

**Affiliations:** 1 Radiology, Drexel University College of Medicine, Philadelphia, USA; 2 Cardiothoracic Surgery, Drexel University College of Medicine, Philadelphia, USA; 3 Radiology, Florida State University College of Medicine, Pensacola, USA; 4 Molecular Biology & Interprofessional Chronic Care, Drexel University College of Medicine, Philadelphia, USA

**Keywords:** abdominal ct, diagnosis of acute appendicitis, imaging in appendicitis, laparoscopic appendectomy, radiology & imaging, ultrasound efficacy in appendicitis

## Abstract

Background

Acute appendicitis is one of the most common surgical emergencies affecting people worldwide. Current guidelines suggest imaging with an immediate computed tomography (CT) scan as the gold standard imaging pathway. However, emerging research has suggested that the addition of an ultrasound (US) can play a role in the diagnosis of acute appendicitis. In this study, we wanted to see if US as an adjunct to the existing imaging pathway of a CT scan had any improvement in 30-day outcomes in adult obese patients.

Method

Using data gathered from real patients using the TriNetX Global Collaborative Network, we conducted a retrospective cohort study on adult obese patients (BMI ≥ 30) with acute appendicitis who were diagnosed by US, with or without a CT scan, versus those who received only a CT scan. In order to balance for possible confounding variables, propensity score matching (1:1) was used to match for common demographic information and comorbidities known to worsen surgical outcomes (hypertension, diabetes mellitus, BMI). The study's primary outcome was emergency room revisit rate after their initial visit. Secondary outcomes included peritoneal abscess, perforation, and peritonitis. All outcomes were measured at 30 days after the acute appendicitis diagnosis.

Results

After propensity score matching, 968 patients were deemed eligible for analysis, with 484 patients within each cohort. There were no detectable statistical differences between these groups after matching for patient demographics and comorbidities. Those who underwent a US did not show significant differences in outcomes compared to those who received a CT scan only. The rates of emergency room revisits were similar between the US and CT scan groups, at 11.98% and 13.64%, respectively (RR = 0.879, p = 0.44). For secondary outcomes, no statistical difference was found for the risk of peritoneal/abdominal abscess (RR = 0.69, p = 0.16), perforation (RR = 1.32, p = 0.35), or peritonitis (RR = 0.90, p = 0.59).

Conclusion

Our study demonstrated no statistically significant difference in outcomes for adult obese patients diagnosed with acute appendicitis who received an ultrasound, compared to those who received a CT scan only.

## Introduction

Acute appendicitis is one of the most common surgical emergencies affecting people worldwide [1]. In the United States, current guidelines suggest imaging to confirm the diagnosis before proceeding with treatment [2]. A computed tomography (CT) scan is the gold standard for diagnostic imaging in adult patients, with the highest sensitivity (>80%) and specificity (>93%) and lower rates of false negatives compared to ultrasound (US) [2].

One of the most important prognostic factors in acute appendicitis is prehospital delay and time to surgery [3]. CT scans are relatively quick compared to other imaging modalities [4]. However, both of these imaging studies can be expensive, and US is an even cheaper and faster imaging alternative [5].

US is widely used for diagnosing acute appendicitis in the pediatric population to avoid radiation exposure from a CT scan while still having effective diagnostic ability [6]. One prior study showed that early US in acute appendicitis avoided the need for a CT scan in most patients and can reduce the time to surgery if it is diagnostic; however, it can also worsen outcomes and delay time to surgery if it is non-diagnostic [7]. This can be further complicated when considering other factors that affect the diagnostic accuracy of US imaging, such as obesity. In one prior study, it was found that children with obesity had significantly higher false negatives with US compared to a non-obese control group, and as a result, the obese cohort had much worse postoperative outcomes [8].

The prior literature on this topic of using US in obese populations with suspected acute appendicitis has primarily focused on the pediatric population, since ultrasonography is currently the first-line imaging tool. However, as US is adopted in more medical centers as a possible first choice in early appendicitis, there exists a research gap if an US is an effective tool in the adult obese population and can have downstream effects on clinical outcomes.

In this study, we wanted to see if US had any use as an imaging modality in obese adult patients. The aim of this study is to compare outcomes in obese adults presenting with acute appendicitis who underwent US, with or without a CT scan, versus those who received only a CT scan.

## Materials and methods

We performed a retrospective cohort study using the TriNetX Global Collaborative Network [9], a federated database of de-identified electronic health records from multiple healthcare organizations. The dataset was current as of March 11, 2026, and contained clinical data (diagnoses, procedures, medications, labs, genomics) from approximately 150 million patients in the electronic health records (EHRs) of 169 healthcare systems. Our analysis was limited to adult patients (≥18 years) with acute appendicitis (ICD-10-CM code K35) [10].

The obesity cohort was created based on the BMI calculated by TriNetX using the TriNetX-curated code TNX 9083 and occurring within one day before or on the index date. The index date is defined as the date of the first diagnosis of acute appendicitis. Patients were assigned to US and CT-only cohorts based on imaging studies occurring within seven days prior to or after the index date. The US cohort was created by combining the Current Procedural Terminology (CPT) codes 76705 for a limited abdominal scan and 76700 for a complete abdominal US and the SNOMED code 363335003 for the US studies by site [9]. A patient with both US and CT coding during the window was classified in the US group regardless of order of imaging. As a result, imaging order could not be established due to limitations of the TriNetX platform, so it is possible US was ordered before or after a CT scan or other imaging modality. The CT-only cohort was created by combining the index date and the ICD-10-PCS code BW2 for a CT scan and by excluding any US codes that occurred within the study window (Appendix).

Data for demographics, weight, height, and comorbid conditions were extracted from the EHRs and identified using ICD-10-CM codes for essential hypertension (I10), type 2 diabetes mellitus (E11), prediabetes (R73.03), hyperglycemia (R73.9), chronic kidney disease (N18), chronic ischemic heart disease (I25), and heart failure (I50). Additionally, 1:1 propensity score matching was performed using a nearest-neighbor algorithm within the TriNetX EHRs to balance the demographic and comorbid covariates. TriNetX uses greedy 1:1 nearest-neighbor matching without replacement with a 0.1 pooled standard-deviation caliper. The methodology used in this study is consistent with TriNetX publishing guidelines [9]. However, significant preexisting conditions such as past abdominal history, pregnancy history, or prior history of appendicitis could not be accounted for, so significant confounding may still exist even after propensity score matching.

The primary outcome was 30-day emergency revisit rate, which was defined as an emergency department revisit within 30 days of the index event. Secondary outcomes included peritoneal abscess (K65.1), appendiceal perforation without abscess (K35.32), and generalized peritonitis (K35.2), occurring within 30 days of diagnosis. All outcomes had to be reported after the index diagnosis of acute appendicitis; any diagnoses prior to the index event were excluded. This analysis was performed within the TriNetX platform, and the outcomes were reported as risk ratio (RR) with 95% confidence intervals (CI) and absolute risk reduction (ARR). A two-sided p-value of < 0.05 was considered to be statistically significant. These outcomes were chosen to evaluate how a US in the diagnosis of appendicitis can influence downstream events, whether that be for improved accuracy and precision, or earlier diagnosis and quicker time to the operating room.

This retrospective study is exempt from informed consent and IRB approval. The data reviewed are a secondary analysis of existing data, do not involve intervention or interaction with human subjects, and are de-identified per the de-identification standard defined in Section §164.514(a) of the HIPAA Privacy Rule. The process by which the data are de-identified is attested to through a formal determination by a qualified expert as defined in Section §164.514(b)(1) of the HIPAA Privacy Rule [11].

## Results

Study population and baseline characteristics

Before propensity score matching, the initial cohorts consisted of 5,653 adult obese patients in the US group and 540 adult obese patients in the CT-only group. Following 1:1 matching to account for potential confounding variables, a total of 968 patients were identified and deemed eligible for analysis. These patients were evenly divided into two matched cohorts: a US group (with or without subsequent CT) containing 484 patients and a CT-only group containing 484 patients.

Propensity score matching (Figure 1) produced demographic and clinical covariates that were balanced between the two cohorts, and there were no statistically significant differences between the cohorts for any of the covariates (all p > 0.05) (Table 1). The mean age at index event was 48.5 ± 17.9 years in the ultrasound cohort versus 48.4 ± 18.1 years in the CT-only cohort (p = 0.916). The gender distribution was also similar, with 235 (48.6%) males in the ultrasound cohort versus 237 (49.0%) in the CT-only cohort.

**Figure 1:**
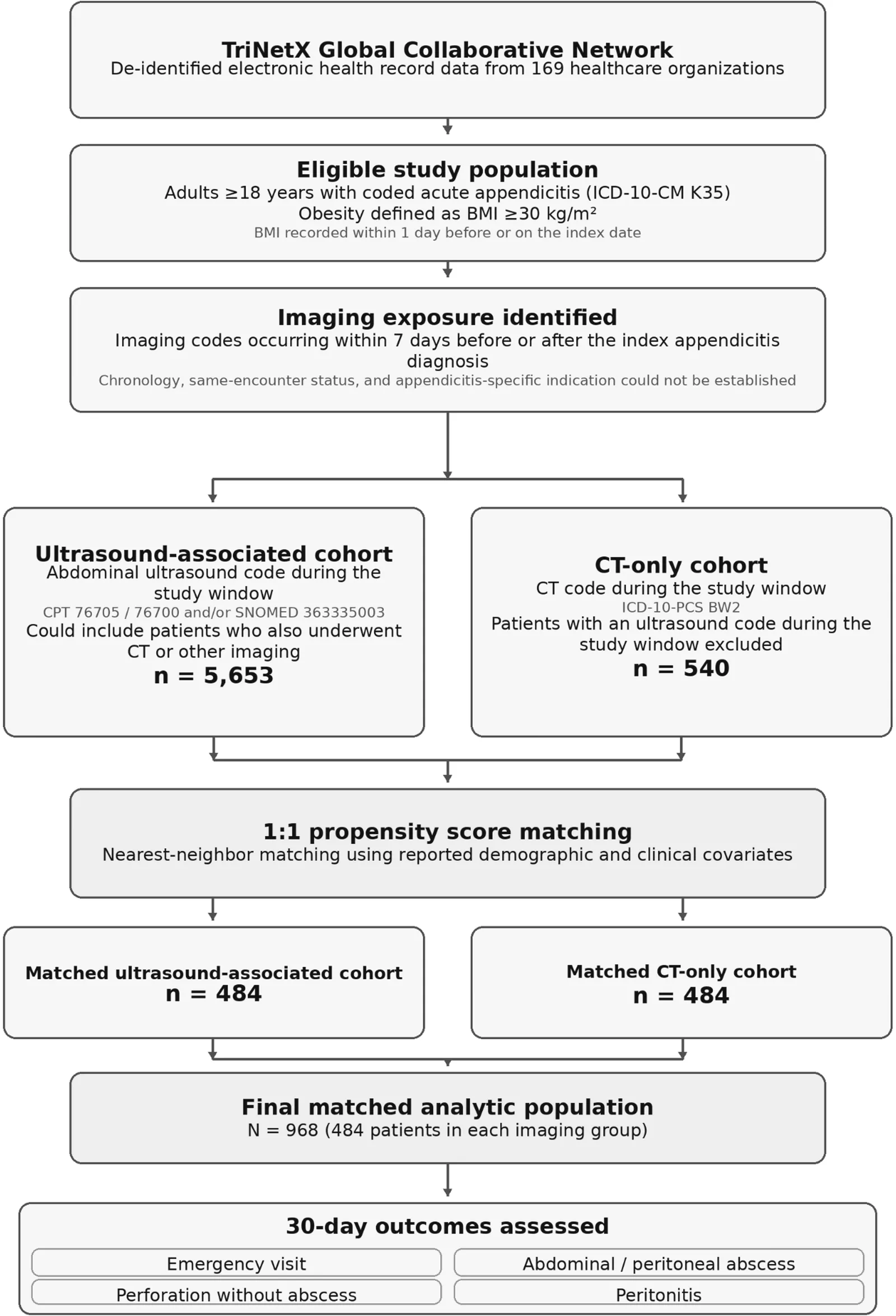
Cohort Selection and Propensity Score Matching Flow Diagram

**Table 1:** Demographics and Clinical Characteristics After Propensity Score Matching of Patients Diagnosed With US or CT-Only A p-value of less than 0.05 was considered statistically significant. *One patient had an unknown gender and, due to the small sample size, could not able to be included in the statistical analysis. **Patients are able to identify as more than one race, so the cohorts in the race category add up to more than the actual cohort number.

Parameters	Ultrasound (N = 484)	CT only (N = 484)	P-Value	Standardized Mean Differences
Demographic				
Age at Index (Mean ± SD)	48.5 ± 17.9	48.4 ± 18.1	0.916	0.007
Sex*				
Male	235 (48.6%)	237 (49.0%)	0.898	0.008
Female	248 (51.2%)	247 (51.0%)	0.949	0.004
Ethnicity				
Not Hispanic or Latino	236 (48.8%)	239 (49.4%)	0.847	0.012
Hispanic or Latino	57 (11.8%)	63 (13.0%)	0.558	0.038
Unknown Ethnicity	191 (39.5%)	182 (37.6%)	0.552	0.038
Race**				
White	235 (48.6%)	231 (47.7%)	0.797	0.017
Black or African American	45 (9.3%)	42 (8.7%)	0.736	0.022
Asian	11 (2.3%)	10 (2.1%)	0.825	0.014
American Indian or Alaska Native	10 (2.1%)	10 (2.1%)	1	<0.001
Native Hawaiian or Other Pacific Islander	0 (0%)	0 (0%)	-	-
Other Race	10 (2.1%)	10 (2.1%)	1	<0.001
Unknown Race	184 (38.0%)	192 (39.7%)	0.598	0.034
Comorbidity				
Essential hypertension (I10)	158 (32.6%)	163 (33.7%)	0.733	0.022
Chronic ischemic heart disease (I25)	47 (9.7%)	41 (8.5%)	0.502	0.043
Prediabetes (R73.03)	10 (2.1%)	10 (2.1%)	1	<0.001
Hyperglycemia, unspecified (R73.9)	12 (2.5%)	10 (2.1%)	0.666	0.028
Type 2 diabetes mellitus (E11)	79 (16.3%)	75 (15.5%)	0.725	0.023
Chronic kidney disease (N18)	28 (5.8%)	26 (5.4%)	0.779	0.018
Heart failure (I50)	24 (5.0%)	33 (6.8%)	0.219	0.079
Laboratory				
BMI (Mean ± SD)	34.2 ± 6.1	34.2 ± 6.6	0.875	0.012

Key comorbidities that could potentially affect outcomes were adequately matched in the study population. The prevalence of the most common comorbidities, such as essential hypertension, type 2 diabetes mellitus, and chronic ischemic heart disease, was 158 (32.6%) vs 163 (33.7%) (p = 0.733), 79 (16.3%) vs 75 (15.5%) (p = 0.725), and 47 (9.7%) vs 41 (8.5%) (p = 0.502) in the two cohorts, respectively. In accordance with the inclusion criteria, both groups were classified as obese with an identical mean BMI of 34.2 (p = 0.875). A love plot was created showing all demographic data below the set caliper of 0.10 standard deviations (Table 2). 

**Figure 2:**
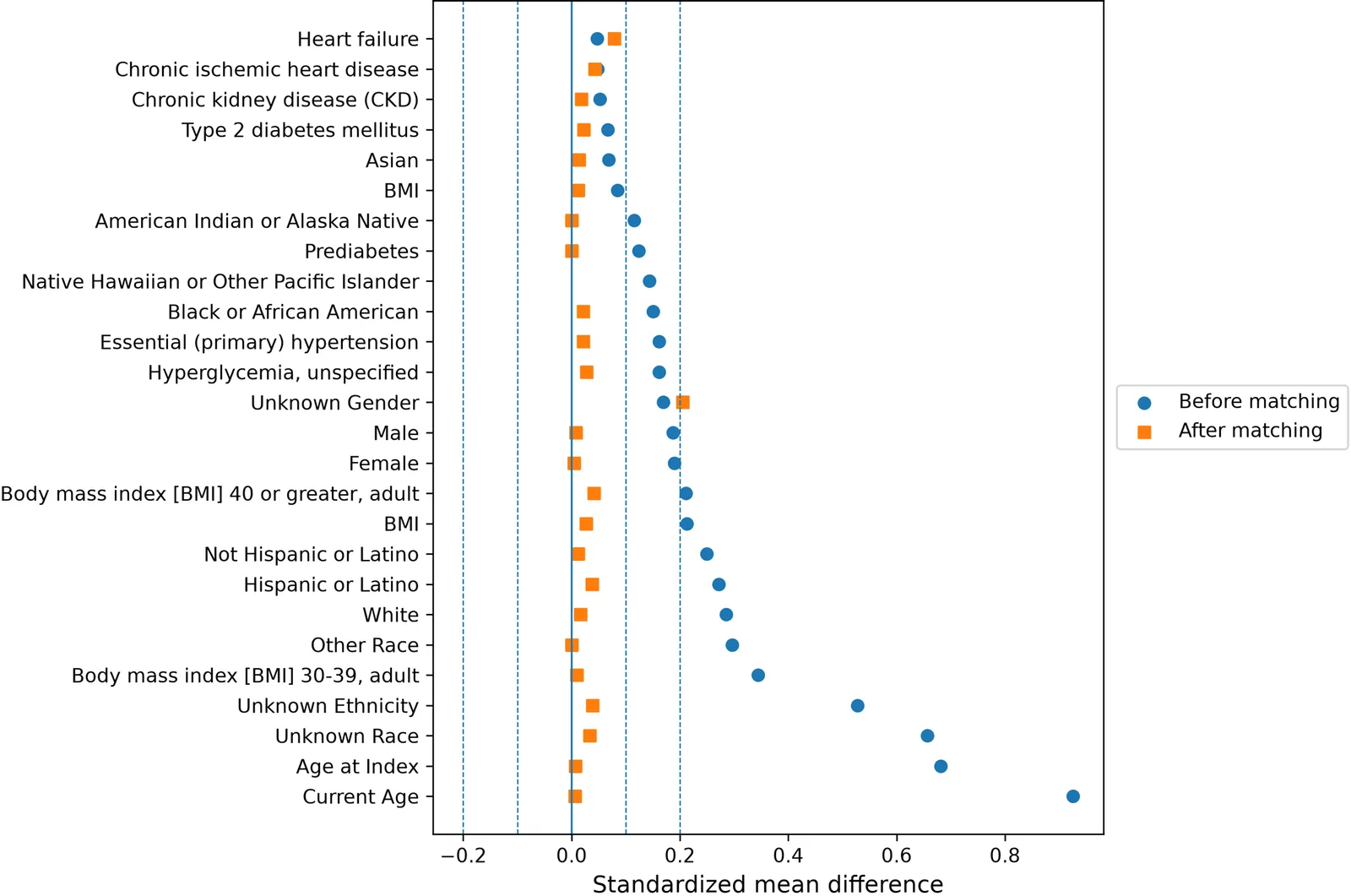
Love Plot of Covariate Balance Before and After Matching

Primary outcome

Analysis of the primary outcome revealed no statistically significant difference in 30-day emergency revisit rates between the two imaging pathways (Table 2). When comparing the emergency revisit rates of the two cohorts, the risk ratio was 0.88 (95% CI: 0.63-1.22; p = 0.442). The number of emergency revisits for the ultrasound vs CT-only cohorts was 58 (12.0%) vs 66 (13.6%).

**Table 2:** Acute Appendicitis Outcome Differences Between US and CT-Only A p-value of less than 0.05 was considered statistically significant. *TriNetX database uses an internal classification code system that does not have a corresponding ICD-10 code for emergency visits

Outcome	US (N = 484)	CT (N = 484)	P-value	Risk Ratio	95% CI	Absolute Risk Difference
Emergency revisit*	58 (12.0%)	66 (13.6%)	0.442	0.88	(0.63-1.22)	-1.6
Abdominal abscess (K65.1)	22 (4.5%)	32 (6.6%)	0.161	0.69	(0.41-1.17)	-2.1
Perforation without abscess (K35.32)	25 (5.2%)	19 (3.9%)	0.355	1.32	(0.73-2.36)	1.3
Peritonitis (K35.2)	44 (9.1%)	49 (10.1%)	0.586	0.9	(0.61-1.32)	1.0

Secondary outcomes

The 30-day secondary outcomes were also not statistically different between the two groups. The number of abdominal/peritoneal abscesses was lower in the ultrasound group at 22 (4.5%) vs. 32 (6.6%) in the CT group but did not reach a statistically significant difference (RR = 0.69; 95% CI: 0.41-1.17; p = 0.161).

The number of appendiceal perforations without abscess was slightly higher in the ultrasound cohort at 25 (5.2%) versus the CT-only cohort at 19 (3.9%), but this finding did not approach statistical significance either (RR: 1.32; 95% CI: 0.73-2.36; p = 0.355). The number/rate of generalized peritonitis was also comparable in both cohorts at 44 (9.1%) in the ultrasound cohort vs 49 (10.1%) in the CT-only cohort (RR: 0.90; 95% CI: 0.61-1.32; p = 0.586).

Using ultrasound as part of the imaging workup in adult obese patients with acute appendicitis did not show a statistically significant difference in 30-day emergency revisit rate or other post-diagnosis complications when compared to using CT as the only imaging modality.

## Discussion

US imaging is an extremely common imaging modality used for the diagnosis of a variety of clinical conditions and offers a variety of benefits, including being readily available and affordable, with a quick diagnostic time [12]. In emergency care, point-of-care US can support rapid bedside decisions, although its diagnostic performance and time advantage remain dependent on the clinical application, selected technique, and operator expertise. Given the highlighted advantages of US scans, we wanted to investigate if this imaging modality could be added to the initial imaging pathway for adult obese patients as part of the diagnostic workup process of acute appendicitis.

In our study, we found that US, when added as an initial imaging modality to the imaging pathway for the workup of acute appendicitis in obese adult patients, showed statistically similar outcomes compared to the CT-only group. We did not observe an associated statistical difference for any of our primary or secondary outcomes after performing propensity score matching to adjust for possible confounding variables, such as hypertension, type 2 diabetes, age, gender, and BMI.

When looking at the primary outcome, emergency revisit rate, we noted an overall rate of 58 (12.0%) in the US group, versus 66 (13.6%) in the CT-only group. While there was a slight improvement in the emergency revisit rate in the US group, it was not statistically significant, with a relative risk ratio of 0.88 (95% CI: 0.63-1.22; p = 0.442). Similarly, secondary outcomes showed statistically similar results for abdominal/peritoneal abscesses (RR = 0.69; 95% CI: 0.41-1.17; p = 0.161), appendiceal perforation (RR: 1.32; 95% CI: 0.73-2.36; p = 0.355) and generalized peritonitis (RR: 0.90; 95% CI: 0.61-1.32; p = 0.586).

The lack of significant statistical difference may be explained by various factors. One major barrier limiting US efficacy in the visualization of internal organs remains high BMI [13]. Prior studies assessing the effectiveness of US in obese children with acute appendicitis found higher rates of inconclusive or inaccurate US studies in the obese group, compared to the non-obese group (OR: 1.86, 95% CI: 1.28, 2.70; p = 0.001), particularly in obese males (OR: 2.55, 95% CI: 1.53, 4.24; p = 0.0003) [14].

Another possible explanation is the limited effectiveness of US in the adult population compared to children. The initial imaging modality in pediatric populations with acute appendicitis is commonly US imaging due to the concerns for radiation exposure in children, with CT studies being reserved for those with an inconclusive US study [15,16]. One prior retrospective cohort study on diagnosing acute appendicitis in the pediatric population found that a definitive interpretation could be made in 72% of studies, with an accuracy of 96% in those reports [17]. Previous meta-analyses on US effectiveness in diagnosing acute appendicitis found that lower sensitivity of 83-83.1% in adults (95% CI: 78%, 87%; 70.3%, 91.1%), compared to children with a sensitivity of 88-91.3% (95% CI: 83.8%, 95.5%; 86%, 90%) [18,19]. The specificity between the two populations was also lower in adults, with the estimated specificity for adults being 90.9% (95% CI: 59.3%, 98.6%), compared to 95.2% in children (95% CI: 91.8%, 97.3%). Another study that looked at the diagnostic accuracy of US in diagnosed acute appendicitis, aimed primarily at children but included those up to the age of 30 years, found age to be an independent risk factor for inconclusive results [20].

The mechanism behind why US is less effective in diagnosing acute appendicitis in adults is not well explored in the current literature. It is hypothesized that it may be related to pediatric populations having a lower BMI on average, with less adipose tissue and subcutaneous gas facilitating better acoustic windows with improved visualization of the appendix [21]. Although improved diagnostic accuracy of US in diagnosing appendicitis is less likely the driving factor behind its use in pediatric populations, but rather an effort to limit ionizing radiation during the childhood development period [22].

Ultimately, our population of adult obese patients had several factors that may explain why US did not significantly change the outcomes, but these results should be interpreted cautiously and as hypothesis-generating.

Strengths and limitations

Our study demonstrated several strengths but did also have multiple limitations in the methodology. To start, this was one of the first studies to look at how US may affect outcomes in adult obese patients with acute appendicitis. Prior studies focused mostly on the pediatric population, with few looking specifically at obese adults. Given the high prevalence of both acute appendicitis as a surgical emergency and obesity as a global epidemic according to the World Health Organization, this topic is timely and addresses an important gap in the literature. The results of the study, while not demonstrating statistically significant differences in outcomes between the two cohorts, can still serve as a first step in attempting to answer the clinically relevant question of whether US has a role as an imaging modality for diagnosing acute appendicitis in adult obese individuals. This study also contributes to the continued improvement and research into the overall optimization of imaging pathway guidelines in the management of acute appendicitis.

The use of TriNetX, a large national database that aggregates real-world clinical data on millions of patients from a variety of different healthcare institutions, was an advantage for completing this study. It allowed us to quickly analyze large data sets and provided a diverse patient set that was not limited by geographical or site-specific institutional practices. Additionally, TriNetX has built-in statistical analysis tools that allowed us to use propensity score matching to improve the internal validity of our study by adjusting for possible confounding variables. We were able to match for common demographic data, such as age, gender, BMI, and race, as well as for comorbidities that may influence postoperative outcomes, such as hypertension, type 2 diabetes mellitus, and chronic kidney disease, all of which could serve as potential confounders. However, it should be noted that propensity score matching cannot guarantee elimination of all confounding variables, such as past surgical history or medication usage, so a residual bias may remain even after matching. While TriNetX is a large database with a significant amount of patient information, our population size was only modest, with 968 patients, or 484 patients in each cohort.

However, while the TriNetX database is large, complete 30-day follow-up across participating healthcare organizations could not be definitively confirmed. It is possible patients may have received subsequent care from institutions outside of TriNetX's reach, and such encounters or outcomes may not have been captured in our data. Similarly, healthcare organization-level clustering could not be accounted for in the analysis, so unmeasured institutional variation could have influenced imaging selection and studied outcomes.

Since this study was a retrospective cohort study, the results should be interpreted as hypothesis-generating and do not offer any new causative evidence, only possible associations. The nature of retrospective cohorts inherently lends itself to selection bias, as the patients were not randomly assigned to the different imaging pathway groups. As a result, clinicians may have preferentially selected initial imaging based on perceived symptom severity, surgical difficulty, and urgency, influencing the measured outcomes. On top of that, the aggregated data pulled from TriNetX relies on the accuracy of correct clinical documentation in the electronic health record. For the initial diagnosis of acute appendicitis, matched comorbidities, and the measured outcomes, we relied on ICD-10 codes [10] that are subject to possible misdiagnosis or mislabeling errors. Since we cannot guarantee the accuracy of these codes, this limits the interpretability of our results. Additionally, ICD-10 and administrative codes lack granular clinical detail that can significantly influence imaging indication, such as clinical presentation, timing of symptom onset, or severity and progression of the disease based on clinician assessment or physical history, which can all affect imaging selection and efficacy.

Furthermore, we could not adjust for factors that could determine imaging selection, such as imaging availability or institutional protocols. As a result, substantial unmeasured confounding remains an issue, and the results cannot definitively comment on the comparative safety between imaging pathways. Ultrasonography is also a much more operator-dependent technology compared to CT scans. This can result in variability of imaging results based on differences in technique and experience level, with a previous study showing significant differences in sensitivity of US in diagnosing appendicitis depending on whether the US was performed by a radiologist versus a non-radiologist healthcare professional [23]. We were not able to account for this within our study, so this may be present as a possible bias in our results. Additionally, because we used the broad ICD-10 code for CT imaging, which is less precise than the subcode for CT of the abdomen and pelvis, we could not differentiate between contrast-enhanced and noncontrast CT imaging. This limits the specificity of the imaging codes for acute appendicitis and introduces the possibility that patients underwent imaging for other comorbidities unrelated to their diagnosis, including other abdominal pathologies.

Finally, while this study focused on comparing the use of US versus CT-only for the workup of acute appendicitis, the exact order of imaging studies could not be controlled for due to limitations of the TriNetX pathway. As a result of this broad imaging timeline, it permits the possibility that an imaging examination occurred after the index diagnosis or was performed for an indication unrelated to appendicitis. For instance, our study data on the US cohort only guarantee that a patient received US, but do not eliminate the possibility of them receiving a CT or MRI either before or afterwards. This was done mostly due to the nature of the study focusing on if an US was helpful in improving outcomes, rather than comparing US efficacy directly against a CT scan. This creates potential exposure misclassification and limits the interpretative value of this study in terms of imaging strategy.

## Conclusions

In this retrospective cohort study with propensity score matching, we looked at adult obese patients with acute appendicitis to see if the addition of US, with or without a CT scan, could improve outcomes compared to a CT-only imaging pathway workup. Our results showed no statistically significant difference in outcomes between the two groups at 30 days for emergency revisit rate, incidence rate of peritonitis, peritoneal/abdominal abscess, or perforation.

These findings should be interpreted cautiously and as hypothesis-generating, given the limitations of the study. We speculate these results could be attributable to increased body habitus or non-pediatric age, which have previously been shown to limit the efficacy of ultrasonography. However, we believe further research in this area in the adult population would be beneficial to further investigate these results and to determine whether the current clinical guidelines on imaging protocol are still best practice.

## Appendices

**Table 3:** Glossary of the Procedure, Outcomes, and Diagnosis Codes Source: Ref [10]

Diagnosis	Code	Role
Acute appendicitis	ICD-10-CM K35	Index diagnosis
Obesity/BMI	TriNetX TNX 9083	BMI ≥30 kg/m²
Limited abdominal US	CPT 76705	Ultrasound exposure
Complete abdominal US	CPT 76700	Ultrasound exposure
Ultrasound	SNOMED 363335003	Ultrasound exposure
CT imaging	ICD-10-PCS BW2	CT exposure
Essential hypertension	ICD-10-CM I10	Matching covariate
Type 2 diabetes mellitus	ICD-10-CM E11	Matching covariate
Prediabetes	ICD-10-CM R73.03	Matching covariate
Hyperglycemia, unspecified	ICD-10-CM R73.9	Matching covariate
Chronic kidney disease	ICD-10-CM N18	Matching covariate
Chronic ischemic heart disease	ICD-10-CM I25	Matching covariate
Heart failure	ICD-10-CM I50	Matching covariate
Emergency visit	TriNetX Internal Code	Primary 30-day coded outcome
Abdominal/peritoneal abscess	ICD-10-CM K65.1	Secondary 30-day coded outcome
Perforation without abscess	ICD-10-CM K35.32	Secondary 30-day coded outcome
Peritonitis	ICD-10-CM K35.2	Secondary 30-day coded outcome
